# Murine Retina Outer Plexiform Layer Development and Transcriptome Analysis of Pre-Synapses in Photoreceptors

**DOI:** 10.3390/life14091103

**Published:** 2024-09-02

**Authors:** Soo-Young Kim, Christine Haewon Park, Bo-Hyun Moon, Gail K. Seabold

**Affiliations:** 1 Neurobiology-Neurodegeneration and Repair Laboratory, National Eye Institute, National Institutes of Health, Bethesda, MD 20892, USA; parkchr@snu.ac.kr (C.H.P.); gail.seabold@nih.gov (G.K.S.); 2 Department of Oncology and Lombardi Comprehensive Cancer Center, Georgetown University Medical Center, Washington, DC 20057, USA; bm366@georgetown.edu

**Keywords:** retina outer plexiform layer, photoreceptor synapse, spherule, pedicle, neural retina leucine zipper, transcriptional regulation, gene expression

## Abstract

Photoreceptors in the mammalian retina convert light signals into electrical and molecular signals through phototransduction and transfer the visual inputs to second-order neurons via specialized ribbon synapses. Two kinds of photoreceptors, rods and cones, possess distinct morphology and function. Currently, we have limited knowledge about rod versus (vs.) cone synapse development and the associated genes. The transcription factor neural retina leucine zipper (NRL) determines the rod vs. cone photoreceptor cell fate and is critical for rod differentiation. *Nrl* knockout mice fail to form rods, generating all cone or S-cone-like (SCL) photoreceptors in the retina, whereas ectopic expression of *Nrl* using a cone-rod homeobox (*Crx*) promoter (*Crx*p*Nrl*) forms all rods. Here, we examined rod and cone pre-synapse development, including axonal elongation, terminal shaping, and synaptic lamination in the outer plexiform layer (OPL) in the presence or absence of *Nrl*. We show that NRL loss and knockdown result in delayed OPL maturation and plasticity with aberrant dendrites of bipolar neurons. The integrated analyses of the transcriptome in developing rods and SCLs with NRL CUT&RUN and synaptic gene ontology analyses identified G protein subunit beta (*Gnb*) 1 and p21 (RAC1) activated kinase 5 (*Pak5 or Pak7*) transcripts were upregulated in developing rods and down-regulated in developing SCLs. Notably, *Gnb1* and *Gnb5* are rod dominant, and *Gnb3* is enriched in cones. NRL binds to the genes of *Gnb1*, *Gnb3*, and *Gnb5*. NRL also regulates pre-synapse ribbon genes, and their expression is altered in rods and SCLs. Our study of histological and gene analyses provides new insights into the morphogenesis of photoreceptor pre-synapse development and regulation of associated genes in the developing retina.

## 1. Introduction

The central nervous system is composed of distinct groups of neurons, which have specific morphologies. Neuronal morphologies represent typical functions and connections, which are controlled by genetic cues and synaptic activity [1,2]. However, there is still more to be learned about the genetic codes that determine the functionality and morphology of each neuron type. The retina belongs to the central nervous system but has fewer types of neurons compared to the brain, providing an easily accessible model for studying mechanisms associated with neuronal differentiation, morphogenesis, synaptogenesis, and plasticity.

The retina possesses highly laminated structures, with three layers of nuclei and two synaptic layers, consisting of five major neuron types: photoreceptor, bipolar, horizontal, amacrine, and ganglion cells. The photoreceptor is the first neuron type, which initiates visual cues and transmits them to the brain via second-order and then ganglion cells, which form the optic nerve. The transmission of visual signals occurs in the retina outer plexiform layer (OPL) by synaptic connections between pre-synapses of photoreceptors and post-synapses in the dendritic fields of over 12 sub-types of bipolar neurons, along with the dendrites and axonal terminals of horizontal cell neurons [3,4].

The mammalian photoreceptors are separated into rods and cones. Rods function under low light conditions, and cones respond to daylight, mediating color vision [5]. Depending on the opsins inside S-, S/M-, or M-cones in the murine retina, different spectral wavelengths of light are detected. Rods and cones differ morphologically in outer and inner segments, nuclear regions, and synapses [6,7]. They have a common pre-synapse structure, called the ribbon, consisting of similar molecules such as ribeye and bassoon. However, the rod pre-synapse terminal (spherule) contains only one ribbon, while the cone pre-synapse terminal (pedicle) contains multiple ribbons inside [8,9,10], indicating more complex connections with different types of bipolar neurons and distinct regions of horizontal neurons to code color vision [11,12]. However, so far, the genetic codes to govern or manage specific development between spherule versus (vs.) pedicle are mostly elusive.

The neural retina leucine zipper (NRL), a basic motif-leucine zipper transcription factor essential for rod cell differentiation, actively commits photoreceptor cells to rod cell fate [13,14,15]. In *Nrl*^-/-^ retina, postmitotic photoreceptor precursors do not develop into rods, producing an excess of S-cones or S-cone-like (SCL) cells [15] with multiple ribbons inside [16], and display electroretinographically cone phototransduction and cone bipolar function [13,15,17,18].

Here, we elucidated the details of the retina OPL development in wild-type (WT) and *Nrl*^-/-^ retinas, using individual photoreceptor labeling to understand photoreceptor synapse development, connections, and plasticity. We examined morphological and histological characterization of developing rods and cones and analyzed pre-synapse development between spherule vs. pedicle. Multi-transcriptome datasets [19,20,21] were used to further categorize NRL-target genes delineating photoreceptor pre-synapse development.

## 2. Materials and Methods

### 2.1. Animals

All studies adhered to the Association for Research in Vision and Ophthalmology (ARVO) Statement for the Use of Animals in Ophthalmic and Vision Research and were approved by the Animal Care and Use Committee of the National Eye Institute. WT C57BL/6J, *Nrl*^-/-^ [15], Nrl promoter-driven green fluorescent protein (*Nrl*p-*GFP*) [14], *Nrl*^-/-^/*Nrl*p-*GFP*, *Cone-rod homeobox* (*Crx*) promoter-driven *Nrl* (*Crx*p-*Nrl*) [13], *clomeleon* (*Clm*)-*GFP* [22], *Clm*-*GFP*/*Nrl*^-/-^, and *Clm*-*GFP*/*Crx*p*Nrl* mice were used. *Nrl*^-/-^/*Nrl*p-*GFP, Clm*-*GFP*/*Nrl*^-/-^, and *Clm*-*GFP*/*Crx*p*Nrl* mice were established by mating for this study. Timed pregnant C57BL/6J and CD1 mice used for in vivo retina electroporation were obtained from Jackson Laboratories (Bar Harbor, ME, USA) and Charles River (Rockville, MD, USA). The total numbers of animals used are listed in Supplementary Table S9.

### 2.2. DNA Construction

Nrl promoter-driven enhanced GFP (*Nrl*p-*EGFP*) [23] and 0.5 kb *S-opsin* promoter-driven *tdTomato* (*S-opsin*p-*tdT*) were used to label rod and cone photoreceptors by in vivo electroporation. Previously, the 2.8 kb mouse *Nrl* promoter (−2734 to +119) was cloned into the *pEGFP-N1* vector [23]. For the construction of *S-opsin*p *tdT*, human Ubiquitin C promoter (*pUB)-tdT* was made from *pUB-GFP* vector [24] by replacing *GFP* with *tdT*, and 0.5 kb S-opsin promoter (−529 to −1) was placed into *pUB-tdT*, removing *pUB* by *Sal*I digestion. The following PCR primers were used to amplify the S-opsin promoter: forward 5′-ggtcgacggcaggatgcagttgtttctgc-3′ and reverse 5′-ccgtcgactcccgcttgggatgccct-3′. The *Nrl* shRNA construct (target sequence: ggtcctgtctctatggaagg) was previously described [25].

### 2.3. In Vivo Electroporation

Electroporation was carried out to label photoreceptors, as previously described [25,26], with minor modifications. Newborn WT CD1, C57BL/6J, and *Nrl*^-/-^ pups were anesthetized by chilling on ice. DNA (1–2 μg/μL) in sterile water containing 0.025% fast green with a total volume of 0.2 μL was injected into the subretinal space by a Hamilton syringe. Electrodes (Harvard Apparatus, Holliston, MA, USA) were placed on either side of the head. Five 80 V pulses (50 ms duration and 950 ms interval) were applied to each mouse.

### 2.4. Immunohistochemistry

Mouse eyes were enucleated and the entire eyeballs were fixed in 4% paraformaldehyde (PFA), or the posterior eyecups were fixed for 30 min. The entire eyes were embedded in agarose and sectioned at 100 μm thickness with vibratome (Leica VT1000S; Leica, Wetzlar, Germany), and the posterior eyecups were cryoprotected in sucrose and embedded in optimal cutting temperature (OCT) compound (Sakura Finetek USA Inc., Torrance, CA, USA) and cut with a cryostat (Thermo Microm HM550; Thermo Fisher Scientific, Kalamazoo, MI, USA). Fluorescent staining of retinal sections and whole mounts was performed as described [27]. The following primary antibodies were used: anti-GFP (Rockland Immunochemicals, Pottstown, PA, USA), anti-DsRed (Rockland Immunochemicals), anti-Ribeye (BD Biosciences Transduction Laboratories, San Jose, CA, USA), anti-Protein Kinase C alpha (PKCα) (Sigma-Aldrich, St. Louis, MO, USA), anti-Calbindin (Calbiochem, La Jolla, CA, USA), anti-Receptor accessory protein 6 (Reep6) [28], anti-Cone arrestin (CAR, Millipore, Billerica, MA, USA), anti-Guanine nucleotide-binding protein alpha subunit (Goα) (Chemicon, Billerica, MA, USA), and anti-M-opsin (Millipore). Relevant secondary antibodies were conjugated with Alexa Fluor 488, 568, 633, or 647 (Life Technologies, Grand Island, NY, USA). Alexa Fluor 594-conjugated Peanut agglutin lectin (PNA) (Invitrogen, Carlsbad, CA, USA) was incubated with secondary antibodies. Images were taken on confocal microscopes (Leica, Zeiss 700, and 780; Leica).

### 2.5. Image Analysis

For area measurement of synapse terminals, OPL confocal images were serially taken by less than 0.5 μm thickness from the middle location of whole mount retinas (See Supplementary Figure S1). The most distinct and largest synapse terminal among the series of terminals of each photoreceptor was chosen, and the area was measured using Imaging J software (versions 1.46 and 1.53). For measurements of OPL thickness, PKCα tips with ribbons, and relative ribbon distribution, the middle location of vibratome-sectioned retinas with optic nerve heads was used. Statistical analyses were performed using Student’s T-test and/or one-way ANOVA (Tukey or Kriskal–Wallis test) using Prism.

### 2.6. Gene Profile Analyses

The published data of flow-sorted photoreceptor transcriptome datasets (Gene Expression Omnibus (GEO) accession # GSE74660) [20] and NRL CUT&RUN-seq (GEO accession # GSE197420) [21] were used for gene profile analyses. Data samples were obtained via the fasterq dump of the SRA toolkit (accessed from 10 February to 2 March 2024; https://github.com/ncbi/sra-tools/). The sequencing reads (from postnatal day P2 to P28 rods and SCLs) from GSE74660 RNA seq was trimmed using Trimmomatic v0.39 [29] with parameter HEADCROP:10, and the P10 bio-replicate reads from GSE197420 NRL CUT&RUN-seq were trimmed with parameter ILLUMINACLIP:(adapter):2:15:4:4: true SLIDINGWINDOW:4:20, similar to the previous study [21], with a small modification. Each sample was aligned to the mouse genome (mm10) using HISAT2 [30], and BAM files were converted using SAMtools [31]. For the RNA seq data, three Count matrixes of reads with 55,487 genes were generated using a reference gtf file of GRCm38.102 by featureCounts: Total, Rod_P4_P14, and SCL_P10_P28. The Count matrixes of Rod_P4_P14 and SCL_P10_P28 were applied to identify differentially expressed genes (DEGs) of interest (adjusted (adj.) *p* < 0.05, fold changes 1.5 or 2) by DESeq2 [32]. The count matrix of Total (P2 to P28 rods and SCLs datasets) was further normalized using the transcript per million (TPM) method, and TPMs were used to generate heatmaps. MACS2 was used to identify peaks of the P10 NRL CUT&RUN-seq data [33], and bamCoverage of deepTools was used to generate bigwig files with parameter normalization using bins per million mapped reads (BPM) [34]. For the gene annotation, a bed file of the combined peaks of 4 P10 bio-replicates of NRL CUT&RUN-seq was generated using MSPC [35] and annotated using the GREAT annotation tool with its basal parameters [36]. The gene lists of RNAseq and NRL CUT&RUN-seq were integrated using the Venn_Diagram webtool (accessed on 25 February 2024; https://bioinformatics.psb.ugent.be/webtools/Venn/), and heatmaps were generated using ggplot2 from R Studio. To identify candidate synapse genes in photoreceptors, Synaptic Gene Ontologies and Annotations (SynGO) [37] and DAVID gene functional classification analyses [38] were used.

## 3. Results

### 3.1. Wild-Type Outer Plexiform Layer Development in Murine Retina

**Figure 1:**
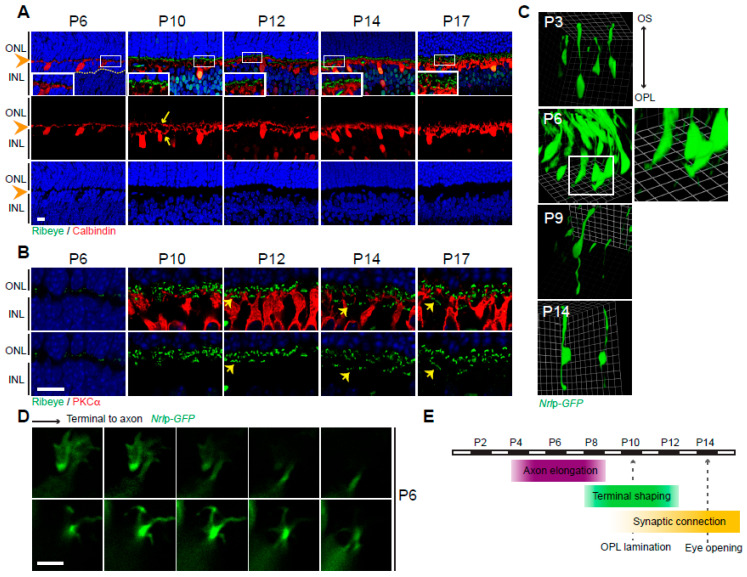
Wild-type outer plexiform layer (OPL) development. (**A**) Developing OPL stained by anti-Ribeye (synaptic ribbons, green) and anti-Calbindin (horizontal neurons, red). Nuclei stained with DAPI. The boxed areas are shown in insets with higher magnification. OPL (arrowheads) and separate fields of dendrites and axon branches from horizontal neurons (arrows) are shown. (**B**) Developing OPL stained by anti-Ribeye (green) and anti-PKCα (rod bipolar neurons, red). Clusters of pedicle ribbons (arrows) are shown. (**C**) Developing rod photoreceptors. Three-dimensional Volocity converted confocal images labeled by in vivo electroporation of *Nrl*p-*EGFP* plasmids. Insert at P6 is shown with higher magnification. (**D**) Growth cone-like structure at P6 terminals of rod photoreceptors. Confocal images displayed at z-thickness of 0.5 μm from synaptic terminal to axon stalk, labeled by in vivo electroporation of *Nrl*p-*EGFP*. (**E**) Schematic summary of OPL and photoreceptor synapse development. Abbreviations: ONL, outer nuclear layer; INL, inner nuclear layer; OPL, outer plexiform layer; DAPI, 4′,6-diamidino-2-phenylindole; P, postnatal day; PKCα, protein kinase C alpha. Scale bars, 10 μm in (**A**,**B**) and 5 μm in (**D**).

Synaptic morphogenesis and connections in murine retinal OPL occur postnatally, and the number of synaptic connections continues to increase up to 3 weeks after birth [39]. The immunofluorescence staining of vertical retina sections using markers for horizontal neurons (Calbindin), rod bipolar neurons (PKCα), and presynaptic ribbon protein (Ribeye) displays the developing lamination pattern of the retina OPL (Figure 1A,B). As previously described [40], we observed the appearance of the OPL at P6 (Figure 1A, arrowheads). The segregation of axon terminals and dendritic fields of horizontal neurons was visible after P10 (Figure 1A, arrows), and ribbons aligned as clusters in the inner portion of OPL (Figure 1B, arrows), showing rod- and cone-laminated synaptic connections with horizontal and bipolar neurons.

We further examined the sequential events of GFP-labeled rod photoreceptors by in vivo electroporation to elucidate pre-synapse development of photoreceptor axon terminals since the gross observation of immunofluorescence staining in the sectioned retinas fails to give information of individual photoreceptor development. P0 or P1 retinas were in vivo electroporated with *Nrl*p-*EGFP* plasmids (Supplementary Figure S1), and the labeled photoreceptors were considered to represent the developing steps of rod photoreceptors because the majority of rods are generated around the time of birth [41,42]. Observations of morphology were made at time points of P3, P6, P9, and P14 (Figure 1C) using confocal images and three-dimensional (3D) visualization using Volocity software (version 6.0; Perkin-Elmer, Wattham, MA, USA). At P3, rods displayed an elongated segment from the cell body towards the outer segment but no distinct structure in the opposite direction towards the OPL to form an axon (Figure 1C). At P6, rod axon terminals were forming, and lamellipodia-like structures were seen at the leading edges in Volocity restoration (Figure 1C, boxed insert) and in the z-stack serial optical image data of confocal microscopy (Figure 1D). The lamellipodia-like structures at the terminal tips had the appearance of growth cones, exploring their path and leading axonal growth at the axonal terminals [42]. The observation of these structures was temporal and disappeared quickly. The lamellipodia structures were no longer observed in the whole retinas of P9, indicating that the rod axons were actively elongating from P3 until before P9. At P9, we observed the typical shape of rod photoreceptors from the top of the outer segment to the tip of axon terminals (Figure 1C). We concluded that rod axons elongated between P3 and P9, and the OPL lamination started occurring around P9 and P10. Maturation is accelerated thereafter via pre-synapse morphogenesis and synaptogenesis (Figure 1E) between photoreceptors and second-order neurons, the horizontal and bipolar cells [43,44].

### 3.2. Characterization of Individual Spherules and Pedicles

**Figure 2:**
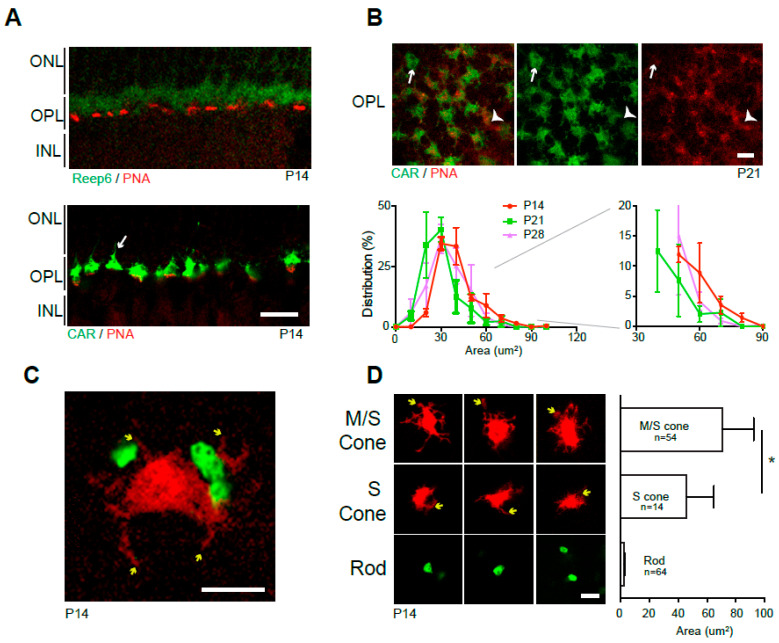
Wild-type spherule and pedicle. (**A**) P14 vertical retina sections stained by anti-Reep6 (spherules; green) and PNA (pedicles; red) (upper panel) or anti-CAR (M-cone, green) and PNA (S-cone, red) (lower panel). (**B**) Horizontal OPL images of retina whole mounts stained by anti-CAR and PNA (upper panels). Pure M-cone (arrows) and S-cone (arrowheads) pedicles are observed. The graph shows the distribution (%, average ± SEM) of CAR pedicle areas in OPL of P14, 21, and 28 whole mount retinas. Over 180 CAR positive pedicles were measured from 3 wild-type C57BL/6J retinas. The area distribution after 30 μm^2^ is magnified on the left side. (**C**) Spherules (green) and a pedicle (red) in CD1 retina whole mount labeled by in vivo electroporation of *Nrl*p-*EGFP* and *S-opsinp-tdT*. Telodendria (yellow arrows) are observed. (**D**) Representative images of M/S-, S-cone pedicles and spherules and their area size comparison. Telodendria (yellow arrows) are observed in cones. M-cone and pure S-cone pedicles are segregated by anti-M-opsin staining in the retina whole mounts labeled by *S-opsin*p-*tdT* electroporation. M/S-pedicles (n = 54), S-pedicles (n = 14), and Rod spherules (n = 64) from 3 to 5 wild-type CD1 retinas were measured. The graph displays the average ± SD of each: 70.79 ± 21.48 μm^2^ for M pedicles, 45.91 ± 18.97 μm^2^ for S pedicles, and 2.64 ± 0.81 μm^2^ for rod spherules. * *p* ≤ 0.05, two-tailed T-test. Abbreviations: CAR, cone arrestin; PNA, peanut agglutin lectin; *S-opsin* promoter-driven *tdTomato (S-opsinp-tdT*); P, postnatal day; ONL, outer nuclear layer; OPL, outer plexiform layer; INL, inner nuclear layer. Scale bars, 10 μm in (**A**)**,** and 5 μm in (**B**–**D**).

To further identify spherules and pedicles in mature OPL, the retina vertical sections were double-labeled with antibodies against spherules (Reep6) [28] and pedicles (PNA) [11] (Figure 2A). Staining displayed segregated labeling of spherules (green) and pedicles (red). Double labeling of PNA and CAR in the sectioned retina (Figure 2A) and the OPL of the whole mount retina (Figure 2B) demonstrated the distribution of S-, M/S-, and M-cone pedicles since the PNA labeling is dominant in S-cone pedicles [45,46] and CAR labels M-cone pedicles [47]. The retina stained with CAR and PNA reveals the dominant pedicle type of M/S-cones, along with a smaller number of pure M- (white arrows) and pure S-cone pedicles (arrowheads) (Figure 2A,B). We determined the size of the pedicles by measuring the CAR-stained areas in the OPL of retina whole mounts and found that the synaptic terminal size reached full size before eye opening around P14 in mouse retinas (Figure 2B).

To clearly distinguish the morphology and size of individual spherules and pedicles, we labeled rods and cones separately by gene delivery of *Nrl*p-*EGFP* (rods) and *S-opsin*p-*tdT (*cones) plasmids. The confocal image in Figure 2C presents representative spherules and a pedicle in their relative size and morphology, and telodendria on cone pedicles are clearly observed (Figure 2C, yellow arrows). We also stained the retinas with M-opsin antibody to discriminate M-cone (M/S) and pure S-cone pedicles (Supplementary Figure S2). The area measurement of synaptic terminals (Figure 2D) indicated that M-cone pedicles were 1.5-fold bigger than S-cone pedicles, consistent with a previous report [45]. Compared to pedicles, the size of rod spherules was tiny, with over a 20-fold size difference.

### 3.3. Characterization of Pre-Synapses in Nrl^-/-^ Photoreceptors

**Figure 3:**
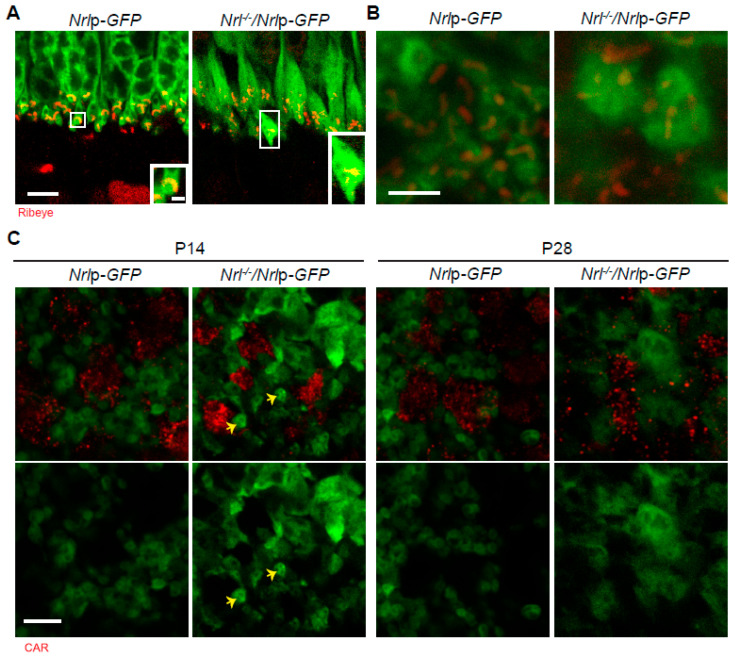
Photoreceptor pre-synapse terminals in *Nrl*^-/-^ retina. (**A**) Vertical retina sections of P18 *Nrl*p-*GFP* and *Nrl*^-/-^/*Nrl*p-*GFP* mice, stained by anti-Ribeye (red). (**B**) Horizontal OPL of retina whole mounts in P18 *Nrl*p-*GFP* and *Nrl*^-/-^/*Nrl*p-*GFP* mice, stained by anti-Ribeye (red). (**C**) Horizontal OPL of retina whole mounts in P14 and P28 *Nrl*p-*GFP* and *Nrl*^-/-^/*Nrl*p-*GFP* mice, stained by anti-CAR (red). Arrows indicate pre-synaptic terminals in small size. Abbreviations: *Nrl*, neural retina leucine zipper; *GFP*, green fluorescent protein; *Nrl*p-*GFP*, *Nrl* promoter-driven *GFP*; P, postnatal day; CAR, cone arrestin. Scale bars, 1 μm in magnified box of (**A**), 5 μm in (**A**,**C**), and 2.5 μm in (**B**).

**Figure 4:**
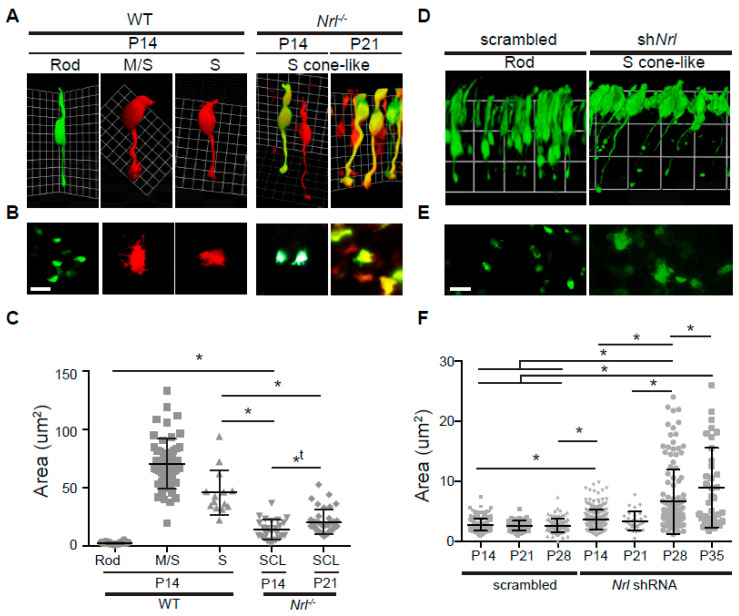
Pre-synapse comparison in wild-type rod, M/S-cone, pure S-cone, and *Nrl*^-/-^ or *Nrl* knockdown S-cone-like (SCL) photoreceptors. (**A**) Representative Volocity 3D images of wild-type rod, M/S-cone, pure S-cone, and *Nrl*^-/-^ SCL photoreceptors, taken from wild-type or *Nrl*^-/-^ retina whole mounts labeled by *Nrl*p-*EGFP*, *S-opsinp-tdT*. Rods (green only), cones (red only) and SCLs (mixed green and red) were imaged. M/S- and pure S-cones were differentiated by staining with an anti-M-opsin antibody. (**B**) Representative confocal images of pre-synapse terminals of wild-type rod, M/S-cone, pure S-cone, and *Nrl*^-/-^ SCL photoreceptors. (**C**) Size distribution of pre-synapses in wild-type rod (n = 64), M/S-cone (n = 54), pure S-cone (n = 14) and *Nrl*^-/-^ SCL (P14, n = 25; P21, n = 38) photoreceptors. (**D**) Representative Volocity 3D images of P28 retina whole mounts labeled by electroporation of scrambled or *Nrl* shRNA plasmid (sh*Nrl*) with *Nrl*p-*EGFP* (2:1 ratio). (**E**) Representative confocal images of pre-synaptic terminals expressing scrambled or *Nrl* shRNA. (**F**) Size distribution of pre-synapses in control (P14, n = 248; P21, n = 64; P28, n = 98) and developing *Nrl* shRNA SCL photoreceptors (P14, n = 246; P21, n = 31; P28, n = 124; P35, n = 36) labeled with *Nrl*p-*EGFP*. Data of area in measurement were analyzed by one-way ANOVA (Tukey or Kriskal–Wallis test) and T-test (two-tailed) in Prism. *, statistically meaningful in one-way ANOVA and T-test; *t, statistically meaningful in T-test. Abbreviations: WT, wild-type; *Nrl*, neural retina leucine zipper; 3D, three-dimensional; *Nrl*p-*EGFP*, *Nrl* promoter-driven enhanced *GFP*; *S-opsin* promoter-driven *tdTomato* (*S-opsinp-tdT*); P, postnatal day; SCL, S-cone-like; shRNA, short hairpin ribonucleic acid. Scale bars, 5 μm in (**B**,**E**).

NRL is a major transcription factor governing rod differentiation [5], and a study showed that the retina OPL of 18-week-old *Nrl*^-/-^ mice has pedicle-like pre-synapses using electron microscopy [16]. We expanded the previous observation by examining the pre-synaptic terminals of GFP-labeled photoreceptors in WT *Nrl*p-*GFP* and *Nrl*^-/-^/*Nrl*p-*GFP* mice (Figure 3) and postnatally delivered *Nrl*p-*EGFP* and/or *Nrl* short hairpin ribonucleic acid (shRNA) in photoreceptors (Figure 4) at different developing stages. We consistently observed enlarged presynaptic terminals of the original rods in P18 retina vertical sections and whole mounts of *Nrl*^-/-^/*Nrl*p-*GFP* mice, compared to those of WT *Nrl*p-*GFP* (Figure 3A,B). The staining of the ribbon structure protein, Ribeye (red), further revealed the presence of several ribbons inside *Nrl*^-/-^ SCL pre-synapses. However, there were also still some tiny spherule-like pre-synapses in the retina OPL of *Nrl*^-/-^/*Nrl*p-*EGFP* mice at P14 (yellow arrows in Figure 3C), suggesting a delayed commitment of the pedicle-like morphologies in SCLs or delayed size maturation in these pre-synapses compared to those in WT retina. To verify this observation in individual pre-synapses as well, we labeled rods and cones with *Nrl*p-*EGFP* and *S-opsin*p-*tdT* plasmids by in vivo electroporation (Figure 4). The SCLs in *Nrl*^-/-^ retinas labeled by both *Nrl*p-*EGFP* and *S-opsin*p-*tdT* plasmids displayed a larger size of pre-synapse terminals than those of WT spherules but were still smaller than WT S-cone pedicles (Figure 4A–C). The SCL pre-synapse terminals also continued to significantly increase in size from P14 (14.17 ± 8.42 μm^2^) to P21 (20.61 ± 10.39 μm^2^) (Figure 4C). There was a similar observation in postnatal *Nrl* knockdown photoreceptors. Postnatal *Nrl* knockdown was applied to the CD1 retinas using *Nrl* shRNA by in vivo electroporation. A 2:1 ratio of *Nrl* shRNA and *Nrl*p-*EGFP* plasmids was applied to knockdown NRL expression because this ratio displayed reduced variability in protein expression. (Supplementary Figure S3). The postnatal *Nrl* knockdown demonstrated an increasing size of SCL pre-synaptic terminals by P35 (Figure 4D–F), but the size of SCL pre-synapse terminals did not reach the size of WT S-cone pedicles (Figure 4C). Meanwhile, rod spherule size was not different from P14 to P28 in the retinas of CD1 mice (Figure 4F). We additionally observed that the pedicle size of the M-cone in *Nrl*^-/-^ retinas was smaller than that of WT M-cone pedicles. The smaller size in the M-cone pedicle was maintained from P14 to P21 (Supplementary Figure S4). Our observation indicates that the pedicle-like morphogenesis in pre-synapses of *Nrl*^-/-^ SCLs is gradually determined and that cells originally committed to rods might be switchable, transforming to SCLs.

### 3.4. Nrl^-/-^ Retina Outer Plexiform Layer Development

**Figure 5:**
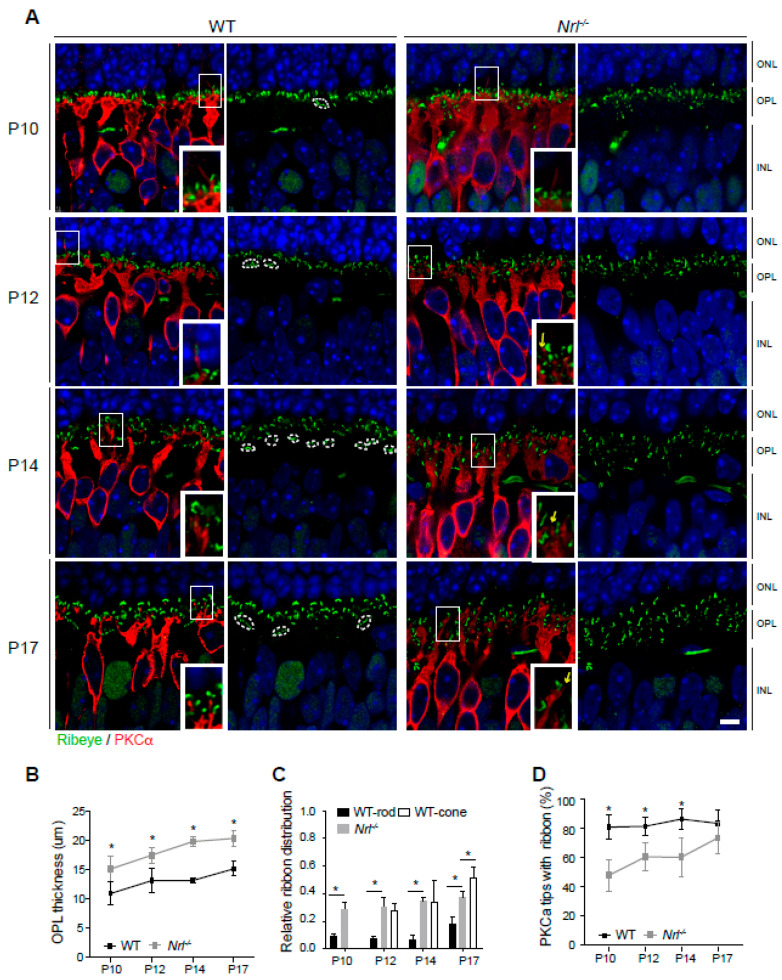
Outer plexiform layer development and synaptic connection in *Nrl*^-/-^ retina. (**A**) Developing (P10 to P17) retinas of wild-type and *Nrl*^-/-^ mice stained by anti-Ribeye (green) and anti-PKCα (red). Clustered pedicle ribbons (white dotted lines) and dendritic tips of rod bipolar neurons without synaptic ribbons (yellow arrows) are observed. (**B**) Comparison of OPL thickness in developing wild-type and *Nrl*^-/-^ retinas. Measurement was quantified on five images of the middle retina (with optic nerve head) from each of three to four animals in different developing stages. Values represent mean ± SD. * *p* ≤ 0.05, two-tailed T-test. (**C**) Comparison of the ribbon distribution in OPL. Distance of ribbon location from the ONL bottom when the OPL thickness is considered 1.0. The location of individual ribbons was measured with each OPL thickness in over two images from each of three to four animals. Values represent mean ± SD. * *p* ≤ 0.05, two-tailed T-test. (**D**) Number comparison (%) of rod bipolar neuron dendritic tips with or without ribbons aligned at their tops. Dendritic tips of rod bipolar neurons were measured at P10 (*WT*, n = 363; *Nrl*^-/-^, n = 627), P12 (*WT*, n = 445; *Nrl*^-/-^, n = 953), P14 (*WT*, n = 433; *Nrl*^-/-^, n = 691), and P17 (*WT*, n = 197; *Nrl*^-/-^, n = 269). Values represent mean ± SD. * *p* ≤ 0.05, two-tailed T-test. Abbreviations: WT, wild-type; *Nrl*, neural retina leucine zipper; P, postnatal day; PKCα, Protein Kinase C alpha; ONL, outer nuclear layer; OPL, outer plexiform layer; INL, inner nuclear layer. Scale bars, 5 μm in (**A**).

To obtain a deeper understanding of developing OPL in *Nrl*^-/-^ retinas, we stained pre-synapse ribbons (Ribeye), rod bipolar neurons (PKCα), and ON-bipolar neurons (Goα) in WT, *Nrl*^-/-^ (cone-only), and *Crx*p*Nrl*, (rod-only) mice at P10 to P17 (Figure 5). There were several differences between WT and *Nrl*^-/-^ retinas during the OPL development (Figure 5A). First, there was the increased thickness of OPL observed in *Nrl*^-/-^ retinas (Figure 5B), and there were longer, extended dendritic stalks of the rod bipolar neurons in *Nrl*^-/-^ retinas (Supplementary Figure S5), consistent with a previous report [48]. Third, there was no clear OPL segregation in *Nrl*^-/-^ retinas, whereas, in the inner portion of WT OPL retinas, clusters of cone ribbons were observed. (Figure 5A, white dashed circles). The ribbon distribution was measured as the relative location within the OPL by numerical assignment from 0 to 1, where 0 corresponds to the location at the border between ONL and OPL and 1 to the location at the border between OPL and INL, as previously reported [49]. The ribbon location of the WT pedicles was not measured at P10 because the clusters of ribbons were not observed in all sections at this stage. However, the spherule and pedicle ribbons started segregating after P10 (Figure 5C). The ribbon locations were measured by the shortest length from the bottom of the ONL (Supplementary Figure S6), and the relative locations were calculated within the OPL. Spherule ribbons were ultimately located within one of the five upper parts of the OPL (0.183 ± 0.046), while pedicle ribbons were at the middle of the OPL (0.518 ± 0.077), similar to our previous report [49]. At P17, the average photoreceptor ribbon location in *Nrl*^-/-^ OPL was between WT spherules and pedicles (0.377 ± 0.044) (Figure 5C). Next, we measured possible synaptic connections of rod bipolar neurons with photoreceptor pre-synapses by counting the number of stained PKCα tips with ribbons at their top since rod bipolar neurons are stained with PKCα antibody [8] (Figure 5A,D). Over 80% of dendritic tips of rod bipolar neurons had ribbons in WT OPL (Figure 5D), while *Nrl*^-/-^ OPL had fewer synaptic connections between photoreceptors and rod bipolar neurons at early stages of development (Figure 5A, yellow arrows). However, by P17, the percentage of ribbon tips aligned with rod bipolar dendritic tops was not significantly different (Figure 5D), although there was still a portion of ribbons located under the borderline of the rod bipolar dendritic fields in *Nrl*^-/-^ OPL, compared to WT (Supplementary Figure S7). Over 20% of the ribbons in *Nrl*^-/-^ retina were located under the top dendritic borderline of the rod bipolar neurons, while under 5% of the ribbons were in WT retina, indicating that less than 5% of the ribbons in the WT OPL of the vertical retina sections belong to the pedicles, whereas at least over 20% of the ribbons in *Nrl*^-/-^ retina do not connect with rod bipolar neurons.

**Figure 6:**
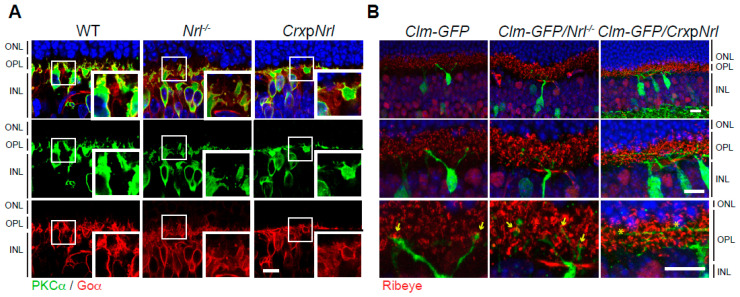
Synaptic plasticity in *Nrl* transgenic retinas. (**A**) 1.5-month retinas of wild-type, *Nrl*^-/-^ (cone-only) and *Crx*p*Nrl* (rod-only) mice stained by anti-PKCα (rod bipolar neurons, green) and anti-Goα (all ON bipolar neurons, red). (**B**) Retinas from 1.5-month-old *Clm-GFP* (type 9 cone bipolar neurons, green), *Clm-GFP*/*Nrl*^-/-^, and *Clm-GFP*/*Crx*p*Nrl* mice stained with anti-Ribeye (red). Abbreviations: WT, wild-type; *Nrl*, neural retina leucine zipper; *Crx*p*Nrl*, *Cone-rod homeobox* promoter-driven *Nrl*; Clm, clomeleon; GFP, green fluorescent protein; PKCα, Protein Kinase C alpha; Goα, guanine nucleotide-binding protein alpha subunit; ONL, outer nuclear layer; OPL, outer plexiform layer; INL, inner nuclear layer. Scale bars, 10 μm in (**A**,**B**).

To further examine the morphological changes in the dendritic fields and connections of cone bipolar neurons caused by disrupted photoreceptor inputs, we stained the retinas of 1.5-month WT, *Nrl*^-/-^ (cone-only), and *Crx*p*Nrl* (rod-only) [13] mice with PKCα (green) and Goα (red) antibodies (Figure 6A). In addition, we stained *Clm-*GFP [22], *Clm-GFP/Nrl*^-/-^, and *Clm-GFP/Crx*p*Nrl* mice with Ribeye (red) (Figure 6B). Goα antibody labels all ON-bipolar neurons, including rod bipolar neurons [50,51], and *Clm-GFP* specifically labels type 9 cone bipolar neurons [45], which connect with S-cone photoreceptors. In the WT retina, dendrites of ON-cone bipolar neurons (PKCα negative and Goα positive) were located clearly in the lower portion of OPL, whereas in *Nrl*^-/-^ and *Crx*p*Nrl* retinas, dendrites of ON-cone bipolar neurons extended to the level of dendritic tips of rod bipolar neurons, indicating that normal OPL lamination was not achieved (Figure 6A). Furthermore, there were differences in synaptic connections between dendritic tips of type 9 cone bipolar neurons and photoreceptor ribbons in WT, *Nrl^-/-,^* and *Crx*p*Nrl* retinas (Figure 6B). The dendritic tips of type 9 cone bipolar neurons in WT retinas were located in the pedicle layer, the lower portion of OPL, while the dendrites of type 9 cone bipolar neurons in *Nrl*^-/-^ retinas were extended into the upper portion of OPL. In *Crx*p*Nrl* mice, horizontally prolonged dendritic branches (Figure 6B, asterisks) of type 9 cone bipolar neurons were observed, suggesting that disturbance of sensory input of photoreceptors leads to dendritic rewiring and synaptic plasticity of second-order neurons in the retina.

Taken together, our data confirm that disturbed input signals modify dendritic fields of connected neurons and might further lead to the rewiring of whole neural circuitries. Furthermore, synapse maturation would be achieved by functional synaptic connections with partner neurons, which may be one reason for the delayed SCL maturation and OPL alteration in these disturbed photoreceptor models.

### 3.5. Comparison of Gene Expression in Spherules vs. Pedicles

**Figure 7:**
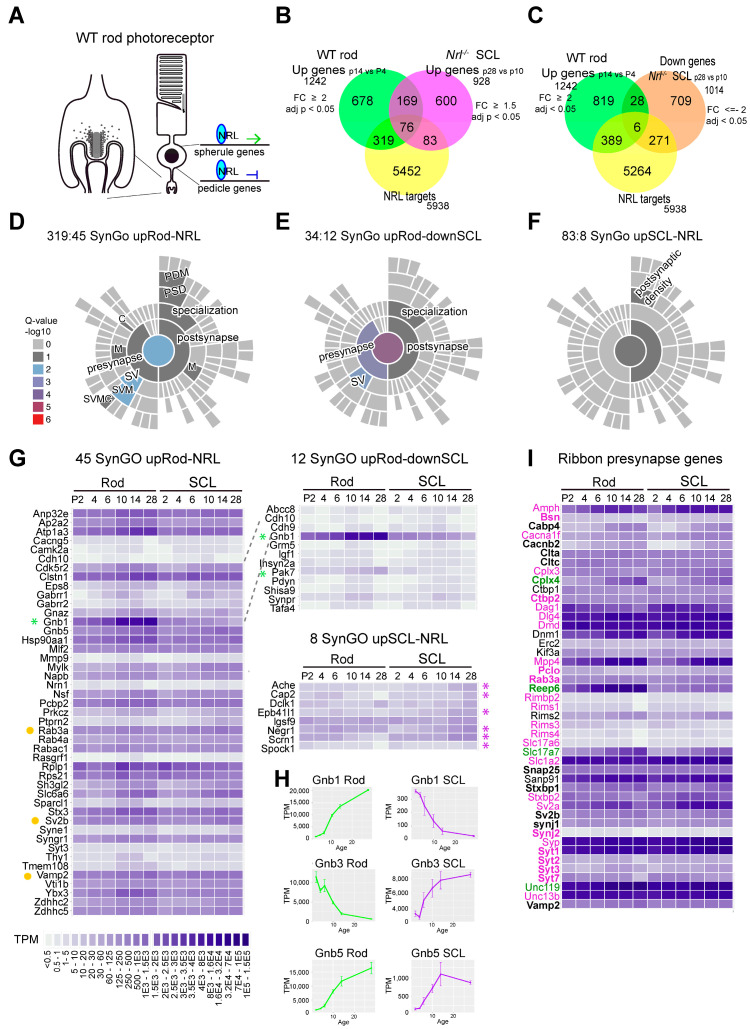
RNA-seq and CUT&RUN-seq analyses of photoreceptor pre-synapse genes. (**A**) Schematic drawing of NRL gene regulation in rod photoreceptors. NRL activates rod genes and suppresses cone genes. (**B**,**C**) Venn diagrams displaying differently expressed genes in rods, SCLs, and NRL-binding genes. (**D**–**F**) SynGO visualizations of upRod-NRL, upRod-downSCL, and upSCL-NRL. (**G**) SynGO synapse genes, in order from P2 to P28 in transcript per million (TPM) heatmaps: SynGO upRod-NRL (left), SynGO upRod-downSCL (upper right), SynGO upSCL-NRL (down right). Genes in which transcripts are upregulated in developing rods and down-regulated in developing SCLs up to P28 are indicated with a green asterisk, and genes in which transcripts are down-regulated in rods and upregulated in developing SCLs are indicated with a purple asterisk. Known ribbon-associated genes are indicated with a yellow dot. (**H**) *Gnb1*, *Gnb3*, and *Gnb5* expression in developing rods and SCLs measured by RNAseq dataset (TPM). The line plots (average ± SD) from all relevant transcripts in 2 to 4 bio-replicates were generated using ggplot2 from R studio. (**I**) Ribbon synapse genes, in order from P2 to P29 in the TPM heatmap. Gene names in magenta correspond to genes showing higher expression in SCLs than rods at P28. Green gene names correspond to genes showing higher expression in rods than SCLs at P28. Gene names in bold font indicate that NRL binds to the genes. Abbreviations: WT, wild-type; *Nrl*, neural retina leucine zipper; upRod-NRL, upregulated NRL binding genes in developing WT rods; upSCL-NRL, upregulated NRL binding genes in developing S-cone-like photoreceptors; downSCL, down-regulated genes in developing S-cone-like photoreceptors; adj. *p*, adjusted *p*-value; TPM, transcript per million; SynGO, synaptic Gene Ontologies and annotations.

Next, to identify synaptic molecules that are specific to spherule vs. pedicle development, we took advantage of the published transcriptome profiles of sorted GFP-labeled photoreceptors in developing *Nrl-GFP* and *Nrl*^-/-^/*Nrl-GFP* retinas [20] and NRL CUT&RUN-seq [21]. NRL regulates genes of both rods and cones; it activates rod genes and suppresses cone genes, directly and/or indirectly, in balance with other transcription factors and epigenetic regulators [5,19,21,52,53] (Figure 7A). NRL directly targets nuclear receptor subfamily 2 group E member 3 (*Nr2E3*), a cone gene repressor, and also binds the promoter region of opsin 1 medium-wave-sensitive (*Opn1mw*) [19]. NRL interactions with both rod and cone genes have been reported [21,54]. Here, in our individual rod cell labeling study (Figure 1C–E), we observed initial segment elongation at P3 and axonal elongation around P6. Synaptic morphology appeared around or before P14 in WT rod photoreceptors, but there was delayed synapse development in SCLs (Figure 3 and Figure 4) and OPL alterations afterward (Figure 5 and Figure 6). Thus, to further understand gene networks in spherules vs. pedicles, we extracted gene lists of upregulated genes in WT rods between P14 vs. P4 (upRod, 2-fold changes, adj. *p* < 0.05) and upregulated genes in *Nrl*^-/-^ SCLs between P28 vs. P10 (upSCL, 1.5-fold changes, adj. *p* < 0.05), and CUT&RUN NRL binding genes in P10 retinas. We then combined the DEGs of upRod and upSCL and the NRL-binding genes using Venn diagrams (Figure 7B). We obtained 319 upregulated NRL binding genes in developing WT rods (upRod-NRL) and 83 upregulated NRL binding genes in *Nrl*^-/-^ SCLs (upSCL-NRL) (Supplementary Table S1). We further integrated the upRod by combining down-regulated SCL genes (downSCL) and obtained 34 genes, of which transcripts are upregulated in developing rods but down-regulated in developing SCLs, regardless of NRL binding (Figure 6C, Supplementary Table S1). We then applied SynGO analyses to these identified genes [37]. SynGO is based on a generic conventional synapse model and only uses synapse genes determined by actual experiments, not based on big data prediction. SynGO analyses identified 45 synapse genes from the 319 upRod-NRL list (Supplementary Table S2), highlighting the Synaptic Vesicle Membrane (SVM) in SynGO hierarchy visualization (Figure 7D), and 12 synapse genes from the 34 upRod and downSCLs with SynGO visualization of pre- and post-synapses (Figure 7E). The well-known post-synapse components in conventional neuronal synapses, for example, postsynaptic density protein 95 (PSD-95; discs large MAGUK scaffold protein 4, Dlg4), dystrophin muscular dystrophy (Dmd), and dystroglycan 1 (DG, Dag1), are pre-synapse ribbon components in the retina [55,56]. There were 8 SynGO genes in the 83 upSCLs-NRL list (Figure 7F) and only 11 SynGO genes from the gene list of 600 upSCLs without NRL binding (Supplementary Table S2), suggesting that SynGO ontology might not entirely represent cone pedicles, a highly specialized sensory neuron synapse. On the other hand, there were 66 SynGO synapse genes in the 678 upRod and no-NRL binding group (Supplementary Table S2). Most of the genes in the 45 SynGO upRod-NRL group also displayed upregulation during SCL development, even if the upregulation did not reach the threshold fold-change (Figure 7G), indicating that there could be common synapse components between spherules and pedicles, even though the expression levels and patterns are different. However, G protein subunit beta (*Gnb*) 1 and p21 (rac family small GTPase 1, RAC1) activated kinase 5 (*Pak5*; also called *Pak7*) transcripts were upregulated in rods from P2 to the P28 but down-regulated in developing SCLs (Figure 7G, indicated by a green asterisk). *Gnb1*, one of the NRL-binding genes, is enriched in rod photoreceptors and associated with rod-cone dystrophy [57]. In our analyses, *Gnb3* and *Gnb5* are also NRL-binding genes (Supplementary Figure S7A,B). *Gnb3* is enriched in developing SCLs, and *Gnb5* is upregulated in developing rods (Figure 7H). *Gnb3* is already known to be expressed in the segments and pedicles of cone photoreceptors and is associated with phototransduction [58,59].

To survey more candidate pedicle and spherule genes, DAVID gene functional classification analyses were applied, and more synapse candidate genes were extracted (Table 1, Table 2 and Table 3 and Supplementary Tables S3–S8). In DAVID analyses of the upRod-NRL genes, there were GOterms such as myelin sheath (GO ID:0043209), neuron projection (GO ID: 0043005), and growth cone (GO ID: 0030426) (Table 1), indicating that our gene analyses properly represent developing rod spherules, including axon elongation. Among them, Neurofascine (Nfasc) is already known as a rod-specific synapse component, and the knockout of *Nfasc* displayed spherule abnormalities and altered rod bipolar dendrites [60]. Previously, we also reported that the knockdowns of *Nfasc*, rod outer segment membrane protein 1 (*Rom1*), Phospholipase C eta 2 (*Plch2*), and Syntrophin alpha 1 (*Snta1*) cause spherule abnormalities [49]. DAVID analyses of upSCL-NRL genes displayed GOterms related to actin binding (GO ID:0003779), actin cytoskeleton (GO ID: 0015629), and microtubule (GO ID:0005874) (Table 2), indicating that pedicles might need additional support or cytoskeleton structuring inside. G protein subunit alpha transducin 2 (*Gnat2*) (GO ID: cell morphogenesis), one of the well-known cone-specific genes, was also identified in the upSCL-NRL gene list. Defects in *Gnat2* cause achromatopsia, or a lack of color vision [61,62]. DAVID analyses of upRod and downSCL added 7 more synapse candidates, as well as 12 SynGo genes (Table 3). Next, we focused on known presynaptic components at photoreceptor ribbon synapses. Synaptic ribbons are specialized presynaptic tethers holding synaptic vesicles and have been described as a conveyor belt to deliver vesicles to the active zone [63]. The differential expressions of several genes, such as Complexin (*Cplx*) 4 and Synaptic vesicle glycoprotein (*Sv*) 2b in spherules and *Cplx3* and *Sv2a* in pedicles, are known [64,65]. In P28 WT and *Nrl*^-/-^ retinas, most ribbon pre-synapse genes (pink indicated in Figure 7I) were expressed higher in SCLs than rod photoreceptors, and just a few genes (complexin4: Cplx4, Receptor accessory protein 6: Reep6, solute carrier family 17: Slc17a7, and unc-119 lipid binding chaperone: Unc119) were highly expressed in rods rather than SCLs (Figure 7I, indicated in green). Interestingly, NRL binds broadly to several pre-synapse ribbon genes (Figure 7I, indicated with bold font).

## 4. Discussion

Our study displays that NRL loss switches and/or alters synapse morphology, size, ribbon number, ribbon location, and connections with bipolar neurons from rods to cones. *Nrl*^-/-^ retinas exhibited pedicle properties in all mature photoreceptors, although the pedicle size in SCL photoreceptors was smaller than that of WT pedicles, and the maturation was delayed compared to that in WT retinas. Furthermore, the switch in photoreceptor presynaptic types caused dendritic alternation of the second-order bipolar neurons in *Nrl*^-/-^ and *Crx*p*Nr*l retinas. However, in the switched photoreceptors, synaptic input or connections did not cause degeneration of the second-order neurons. Therefore, this study provides a good model for studying photoreceptor adaptations and altered synapse development and how altered gene regulation can govern these events.

To identify photoreceptor synapse candidate genes and understand ribbon genes in the context of spherules and pedicles, we applied our synapse development time course in WT and *Nrl*^-/-^ retinas for big data analyses using datasets of developing rods and SCLs [20], NRL CUT&RUN-seq [21], and GO analysis tools, such as SynGO [37] and DAVID [38]. Spherules and pedicles are quite different in their shapes and functions. When over 12 different types of bipolar neurons are considered [66], there might be a variety of synapse alterations and connections among the different types of photoreceptors, from rods to cones and from M-, M/S-, and S-cones. In spite of their differences in morphology and function, there are a limited number of unique genes expressed in spherules vs. pedicles. Reep6 is one gene that is highly expressed only in spherules [67]. Our analyses also revealed *Gnb1* and *Pak7*, whose transcripts were specific in developing and mature rods. Acetylcholinesterase (*Ache*), Cyclase-associated actin cytoskeleton regulatory protein 2 (*Cap2*), Doublecortin-like kinase 1 (*Dclk1*), Erythrocyte membrane protein band 4.1-like 1 (*Epb41l1*), Neuronal growth regulator 1 (*Negr1*), Secerin 1 (*Scrn1*), Sparc/osteonectin, cwcv, and kazal-like domains proteoglycan 1 (*Spock1*) were specific to developing and mature SCLs, suggesting that they could be specific to cone pedicles. Interestingly, Gnb1 was specific in rods, whereas Gnb3 was specific in SCLs. It is already known that Gnb3 is expressed in WT pedicles [58]. However, the synaptic function of Gnb3 is still unknown. Our analyses also suggested that Cplx3 and Sv2a are expressed higher in pedicles than in spherules, and Cplx4 and Sv2b are more specific in spherules. Interestingly, there was differential expression of known and common ribbon pre-synapse genes between rods and SCLs, and NRL regulation of ribbon synapse genes was confirmed (Figure 7I) [54]. However, caution needs to be taken when interpreting the differential expression of pre-synapse ribbon genes between rods and SCLs because SCLs are not WT cones, although current and previous data analyses suggest that SCLs are close to WT cones [68,69]. It is noteworthy that the switched or hybrid photoreceptors in this current, or a previous [16], study did not cause degeneration of the second-order neurons. In addition, *Nrl* knockout delayed pedicle development compared to the WT, and postnatal *Nrl* knockdown using *Nrl* shRNA delayed the switch between spherule vs. pedicle, suggesting that committed photoreceptors might re-enter differentiation and form another type of photoreceptor cell. This finding may be important when considering clinical trials (NCT05203939; NCT04945772; NCT03326336; NCT04278131) using optogenetic or transcription factor gene delivery to treat retinitis pigmentosa or other degenerative retina diseases. Furthermore, the rod or cone photoreceptor synapse genes revealed in this study could be candidate genes for genetic diseases of the brain and sensory system because synapse genes are common components in different neuron sets. For example, our DAVID analyses provide synapse gene lists of Kyoto Encyclopedia of Genes and Genomes (KEGG) DISEASE pathways such as Parkinson’s disease, Huntington’s disease, Alzheimer’s disease, and long-term depression (Supplementary Tables S3–S8). Finally, this study once more indicates that NRL is broadly involved in the regulation of both spherule and pedicle genes during photoreceptor development and paves the road toward a deeper understanding of synapse genes and their function in rod and cone photoreceptors.

## Figures and Tables

**Table 1:** DAVID analysis of Rod upregulated NRL targets. This table displays relevant Gene Ontology (GO) terms and genes that could be associated with synapse development.

GOterm	Go ID	Genes
myelin sheath	0043209	NSF, NAPB, HSP90AA1, ENO1B, ATP1A3, THY1, ENO1, COX6A1, NFASC, PKM, GNB1, GNB5, SLC25A12, ALDOA, SLC25A5
synapse	0045202	RAB3A, NRN1, RPLP1, CABP4, CLSTN1, CAMK2A, ATP1A3, TULP1, YBX3, ZDHHC2, LY6C1, EPS8, GABRR2, GABRR1, SYNGR1, SV2B, TMEM108, RABAC1, SH3GL2, SNTA1, CACNG5, PTPRN2, WHRN, CARTPT, MYRIP, USH2A, CDH10, SPARCL1, RPS21, VAMP2, SNTB2
neuron projection	0043005	HSP90AA1, PACRG, WHRN, CAMK2A, RTN4RL2, LSM4, EPS8, GABRR2, GABRR1, PCP4, NFASC, RIT2, SV2B, BBS7, STX3, VAMP2, CDK5R2, RGS6
dendrite	0030425	GNAZ, RCVRN, KCNJ12, KCNIP2, CAMK2A, ZDHHC5, RTN4RL2, THY1, TRAK1, TXN2, SLC6A6, PEX5L, NFASC, GNB1, TMEM108, STX3
Calcium ion binding	0005509	CRB1, SYT3, RCVRN, KCNIP2, CABP4, CLSTN1, GUCA1A, RHOT2, PCP4, EHD4, VSNL1, PITPNM1, CDH10, CDHR1, SPARCL1, PLCH2, SLC25A12, GAS6, NUCB2, SLC25A25, TGM3
vesicle-mediated transport	0016192	NSF, NAPB, RAB4A, SYT3, TULP1, AP2A2, ARL4D, SPIRE2, STX3, VAMP2, HSPA1B, VTI1B, HSPA1A
ion transport	0006811	KCNG1, KCNJ5, KCNE2, KCNK9, KCNJ12, KCNIP2, SLC6A15, ATP1A3, SLC5A2, SLC4A5, GABRR2, GABRR1, NIPAL1, CNGA1, SCN4A
growth cone	0030426	EPS8, WHRN, ENO1B, RASGRF1, STX3, THY1, ENO1, CDK5R2
calmodulin binding	0005516	PCP4, RIT2, CAMK2A, VAMP2, MYLK, SNTA1, SNTB2
GABA-ergic synapse	0030425	SLC6A6, GABRR2, GABRR1, CDH10, CLSTN1, ZDHHC5
postsynaptic membrane	0045211	SLC6A6, GABRR2, GABRR1, CLSTN1, CACNG5, SNTA1, SYNE1
chemical synaptic transmission	0007268	GABRR2, GABRR1, SV2B, CLSTN1, CARTPT
extracellular matrix	0031012	ADAMTSL1, COL26A1, COL3A1, ADAMTS3, COL4A3, RTN4RL2, MMP9
transmembrane transport	005508	KCNG1, SLC25A29, SV2B, CNGA1, SCN4A, SLC5A2, SLC25A5, SLC25A12, SLC25A25, SLC4A5
cell adhesion	0007155	NFASC, PRPH2, CDHR1, CDH10, CLSTN1, ROM1, COL4A3, ADAM9, SPG7, THY1, CD34
extracellular region	0005576	CRB1, CCDC126, CDNF, PRCD, CLSTN1, LOXL4, TULP1, PLA2G7, GLB1L2, CST3, ADAMTSL1, FTH1, NRTN, FAM3C, CD34, ST3GAL1, CTSB, CHGA, COL26A1, CARTPT, MMEL1, USH2A, MMP9, POMC, COL3A1, RBP3, QPCT, COL4A3, SPARCL1, GAS6, NUCB2, HSPA1A

129 genes: ADAM9, ADAMTS3, ADAMTSL1, ALDOA, AP2A2, ARL4D, ATP1A3, BBS7, CABP4, CACNG5, CAMK2A, CARTPT, CCDC126, CD34, CDH10, CDHR1, CDK5R2, CDNF, CHGA, CLSTN1, CNGA1, COL26A1, COL3A1, COL4A3, COX6A1, CRB1, CST3, CTSB, EHD4, ENO1, ENO1B, EPS8, FAM3C, FTH1, GABRR1, GABRR2, GAS6, GLB1L2, GNAZ, GNB1, GNB5, GUCA1A, HSP90AA1, HSPA1A, HSPA1B, KCNE2, KCNG1, KCNIP2, KCNJ12, KCNJ5, KCNK9, LOXL4, LSM4, LY6C1, MMEL1, MMP9, MYLK, MYRIP, NAPB, NFASC, NIPAL1, NRN1, NRTN, NSF, NUCB2, PACRG, PCP4, PEX5L, PITPNM1, PKM, PLA2G7, PLCH2, POMC, PRCD, PRPH2, PTPRN2, QPCT, RAB3A, RAB4A, RABAC1, RASGRF1, RBP3, RCVRN, RGS6, RHOT2, RIT2, ROM1, RPLP1, RPS21, RTN4RL2, SCN4A, SH3GL2, SLC25A12, SLC25A25, SLC25A29, SLC25A5, SLC4A5, SLC5A2, SLC6A15, SLC6A6, SNTA1, SNTB2, SPARCL1, SPG7, SPIRE2, ST3GAL1, STX3, SV2B, SYNE1, SYNGR1, SYT3, TGM3, THY1, TMEM108, TULP1, TXN2, USH2A, VAMP2, VSNL1, VTI1B, WHRN, YBX3, ZDHHC2, ZDHHC5. SynGO analyses also provided genes underlined.

**Table 2:** DAVID analysis of SCL-upregulated NRL targets. This table displays relevant Gene Ontology (GO) terms and genes that could be associated with synapse development.

GOterm	Go ID	Genes
actin binding	0003779	DIAPH3, PARVG, WIPF1, EPB41L1, MYO7A, CAP2
dendrite	0030425	ACHE, OPN4, NEGR1, NR1D1, IGSF9, CNNM4
actin cytoskeleton	0015629	RINL, PARVG, WIPF1, MYO7A
cell morphogenesis	0000902	GREM1, GNAT2, CAP2
ATP-dependent microtubule motor activity, minus-end-directed	0008569	KIFC1, DNAH7C
axon	0030424	ACHE, OPN4, MAP4, IGSF9, DCLK1
amino acid transmembrane transport	0003333	SLC7A8, SLC38A3
microtubule	0005874	KIFC1, MAP4, DNAH7C
calcium ion binding	0005509	HEG1, CALU, SPOCK1, AMY1, DNAH7C
extracellular region	0005576	GREM1, ACHE, ORM1, CALU, SPOCK1, AMY1

25 genes: ACHE, AMY1, CALU, CAP2, CNNM4, DCLK1, DIAPH3, DNAH7C, EPB41L1, GNAT2, GREM1, HEG1, IGSF9, KIFC1, MAP4, MYO7A, NEGR1, NEGR1, OPN4, ORM1, PARVG, RINL, SLC38A3, SLC7A8, SPOCK1, WIPF1. SynGO analyses also provided genes underlined.

**Table 3:** DAVID analysis of Rod upregulated and SCL down-regulated genes. This table displays relevant Gene Ontology (GO) terms and genes that could be associated with synapse development.

GOterm	Go ID	Genes
synapse	0045202	SYNPR, RNF112, GRM5, CDH10, PDYN, SHISA9, CDH9, INSYN2A
cell-cell junction assembly	0007043	CDH10, NR1H4, CDH9
neuronal dense core vesicle lumen	0099013	IGF1, PDYN
postsynaptic membrane	0045211	GRM5, GRM6, SHISA9, CDH9
glutamatergic synapse	0098978	GRM5, CDH10, IGF1, SHISA9, CDH9
postsynaptic density	0014069	RNF112, GRM5, SHISA9, INSYN2A
calcium-mediated signaling using intracellular calcium source	0035584	GRM5, VCAM1
myotube differentiation	0014902	IGF1, ANKRD2
response to zinc ion	0010043	VCAM1, ABCC8
regulation of long-term neuronal synaptic plasticity	0048169	GRM5, AGT
dendrite	0030425	GRM6, GNB1, PDYN, CDH9
extracellular region	0005576	TAFA4, VCAM1, FRZB, IGF1, PDYN, BMP7

19 genes: *ABCC8*, AGT, ANKRD2, BMP7, *CDH9*, *CDH10*, FRZB, *GNB1*, *GRM5*, GRM6, IGF1, *INSYN2A*, NR1H4, *PDYN*, *RNF112*, *SHISA9*, *SYNPR*, *TAFA4*, VCAM1. SynGO analyses also provided genes underlined. A total of *11 genes* (Italic) showed the highest expression in either central nervous system (CNS) embryonic day18 (E18), cerebellum adult, or frontal lobe cortex compared to other tissues in mouse ENCODE transcriptome data.

## Data Availability

The original contributions presented in the study are included in the article/Supplementary Materials, further inquiries can be directed to the corresponding author/s, and the original data used in this study are already available in NCBI Gene expression omnibus datatbase at accession numbers GES74660 and GSE197420.

## Supplementary Materials

The following supporting information can be downloaded at: https://www.mdpi.com/article/10.3390/life14091103/s1, Figure S1: Retina in vivo electroporation; Figure S2: Pedicle discrimination between M/S and pure S cone photoreceptors; Figure S3: Efficiency of *Nrl* shRNA constructs; Figure S4: Size comparison of cone arrestin; Figure S5:Vertical retina images of WT and *Nrl*^-/-^; Figure S6: OPL thickness and relative ribbon location in WT and *Nrl*^-/-^ retinas; Figure S7: Aberrant synaptic connectivity of rod and cone pre-synapses to distinct biopolar cells; Figure S8: NRL binding to the genes *G protein subunit beta (Gnb)1*, *Gnb3* and *Gnb5*; Table S1: Gene lists of Venn diagram; Table S2: SynGo genes from the gene lists of Venn diagram; Table S3: Upregulated Rod NRL targets—Chart, DAVID; Table S4: Upregulated Rod NRL targets—Clustering, DAVID; Table S5: Upregulated SCL targets—Chart, DAVID; Table S6: Upregulated SCL NRL targets—Clustering, DAVID; Table S7: Upregulated Rod downregulated SCL—Chart, DAVID; Table S8: Upregulated Rod downregulated SCL—Clustering, DAVID; Table S9: Animals used.

## Abbreviations

3D	three-dimensional
adj. *p*	adjusted *p*-value
ARVO	Association for Research in Vision and Ophthalmology
BPM	bins per million mapped reads
CAR	cone arrestin
Clm	clomeleon
Cplx	complexin
*Crx*p*Nrl*	Crx promoter
DAPI	4′,6-diamidino-2-phenylindole
DEGs	differentially expressed genes
Dlg4	discs large MAGUK scaffold protein 4
Dmd	dystrophin muscular dystrophy
downSCL	down-regulated genes in developing S-cone-like photoreceptors
KEGG	Kyoto Encyclopedia of Genes and Genomes
GEO	Gene Expression Omnibus
GFP	green fluorescent protein
Gnb	G protein subunit beta
GO	Gene Ontology
Goα	guanine nucleotide-binding protein alpha subunit
Nr2E3	nuclear receptor subfamily 2 group E member 3
NRL	neural retina leucine zipper
*Nrl*p-*EGFP*	Nrl promoter-driven enhanced GFP
*Nrlp-GFP*	Nrl promoter-driven GFP
OCT	optimal cutting temperature
OPL	outer plexiform layer
P	postnatal day
PFA	paraformaldehyde
PKCα	Protein Kinase C alpha
PNA	peanut agglutin lectin
PSD-95	postsynaptic density protein 95
*pUB*	Ubiquitin C promoter
*S-opsinp-tdT*	S opsin promoter-driven tdTomato
SCL	S-cone like
shRNA	short hairpin ribonucleic acid
Sv	Synaptic vesicle glycoprotein
SynGO	Synaptic Gene Ontologies and annotations
TPM	transcript per million
upRod-NRL	upregulated NRL binding genes in developing WT rods
upSCL-NRL	upregulated NRL binding genes in developing S-cone-like photoreceptors
vs.	versus
WT	Wild-type
