# Supplementary material for: Murine Retina Outer Plexiform Layer Development and Transcriptome Analysis of Pre-Synapses in Photoreceptors
Source: Life (Basel). 2024 Sep 2;14(9):1103. doi: 10.3390/life14091103 (PMC11433150; doi:10.3390/life14091103)
Supplement: Supplementary file 1 [file life-14-01103-s001.zip › Supple figure legends-r2.pdf]

Supplementary Data Figure Legends

Supplementary Figure S1. Retina *in vivo* electroporation. **(A)** Diagram showing subretinal injection and electroporation. **(B)** Representative whole mount image labeled by *Nrlp-GFP*. Labeled photoreceptors located in the middle retina were imaged and measured for the studies.

Supplementary Figure S2. **Pedicle discrimination between M/S and pure S cone photoreceptors. (A-B)** Volocity 3D-processed confocal images (left) and single optic confocal images of segment layer of photoreceptor (top right) and OPL layer (bottom right) in M opsin (blue) stained, *S-opsinp-tdT* (red) electroporated whole retina. Representative M-cone **(A)** and pure S-cone **(B)** photoreceptors.

Supplementary Figure S3. Efficiency of *Nrl* shRNA constructs. Transverse NRL (red) stained outer nuclear layer images of retina electroporated with *Nrlp*-EGFP and sh*Nrl* (ratio 1:1 or 1:2). Relative intensity of NRL signals in retinas electroporated with *Nrlp*-EGFP and sh*Nrl* (ratio 1:1 or 1:2). Scale bar, 5 μm.

Supplementary Figure S4. Size comparison of cone arrestin (CAR) pedicle areas between developing wild type and *Nrl*$^{-/-}$ retinas in a distribution **(A)** and a bar graph **(B)**. 3 retinas of each group were used. In *Nrl*$^{-/-}$ mice, the area measurement of CAR pedicles was limited to those in P14 and P21 retinas, due to unclear boundaries of CAR staining at P28 (WT, P14 n=88, P21 n=188, P28 n=372; KO, P14 n=140, P21 n=66). Values represent mean ± SD.

Supplementary Figure S5. Vertical retina images of WT and *NRL*$^{-/-}$. Whole retina vertical images of WT and *Nrl*$^{-/-}$ retinas (P14) stained by Ribeye (green), PKCα (red) and DAPI. Inserts are twice-magnified. Scale bar, 50 μm.

Supplementary Figure S6. **OPL thickness and relative ribbon location in WT and *Nrl*$^{-/-}$ retinas.** Representative images of DAPI and ribeye-labeled WT and *Nrl*$^{-/-}$ retinas (left panels) and the intensity graphs showing OPL thickness and ribbon location (right panels). OPL thickness was measured by the length between the lowest line of the outer nuclear layer (ONL) and the highest line of the inner nuclear layer (INL). The ribbon OPL locations were measured from the lowest outer nuclear layer (ONL) to the nearest point of ribbon location, and then relative ribbon location in OPL was calculated. Scale bar, 10 μm.

Supplementary Figure S7. **Aberrant synaptic connectivity of rod and cone pre-synapses to distinct bipolar cells. (A)** Graph of the number (%) of ribbons that have extended beyond the borderline of rod bipolar dendritic tips. Four retinas (4 sections per each retina) were used for this measurement (mean ± SEM). **(B)** Representative images stained by PCKα (blue) and ribeye (red) in *Nrlp*-EGFP and *Nrl*$^{-/-}$/*Nrlp*-EGFP retinas. The borderline of rod bipolar dendritic tips is indicated by dotted-line.

Supplementary Figure S8. **NRL binding to the genes *G protein subunit beta (Gnb)1*, *Gnb3* and *Gnb5*. (A)** NRL binding density profiles of Gnb1, Gnb3 and Gnb5. The NRL CUT&RUN-seq density views from P10 biological replicates on bins per

million mapped reads (BPM) were visualized using IGV (2.17.1). **(B)** NRL binding density profiles of Gnb1, Gnb3 and Gnb5. The NRL and CRX ChIPseq peaks (GSE20012) were visualized using reads per million (RPM) by University of California, Santa Cruz (UCSC) genome browser view.
