# Supplementary material for: Murine Retina Outer Plexiform Layer Development and Transcriptome Analysis of Pre-Synapses in Photoreceptors
Source: Life (Basel). 2024 Sep 2;14(9):1103. doi: 10.3390/life14091103 (PMC11433150; doi:10.3390/life14091103)
Supplement: Supplementary file 1 [file life-14-01103-s001.zip › supplemental table S9.pdf]

Table. S9 Animals used.

Species	Mutant forms	Ages	Numbers
B6J	Wild Type	P6	2, Section
		P9	2, Section
		P10	4, Section
		P12	4, Section
		P14	4, Section; 6, Whole
		P17	4, Section
		P21	3, Whole
		P28	3, Whole
	Nrl$^{-/-}$	P10	4, Section
		P12	4, Section
		P14	4, Section; 6, Whole
		P17	4, Section
		P21	6, Whole
		P28	3, Whole
		1.5 month	3, Section
	Nrlp-GFP	P12	4, Section
		P14	4, Whole
		P18	4, Section; 3, Whole
		P28	4, Whole
	Nrl$^{-/-}$*/Nrlp-GFP*	P12	4, Section
		P14	4, Whole
		P18	4, Section; 3, Whole
		P28	4, Whole
	CrxpNrl	1.5 month	3, Section
	Clm-GFP	1.5 month	3, Section
	Clm-GFP/Nrl$^{-/-}$	1.5 month	3, Section
	Clm-GFP/CrxpNrl	1.5 month	3, Section
CD1	Wild Type	P3	2, Whole
		P6	5, Whole
		P9	3, Whole
		P14	16, Whole
		P21	10, Whole
		P28	11, Whole
		P35	3, Whole
