# Supplementary figures and images for: Murine Retina Outer Plexiform Layer Development and Transcriptome Analysis of Pre-Synapses in Photoreceptors
Source: Life (Basel). 2024 Sep 2;14(9):1103. doi: 10.3390/life14091103 (PMC11433150; doi:10.3390/life14091103)

## Supplemental figure S1

### Retina *in vivo* electroporation

**A**

Subretinal injection

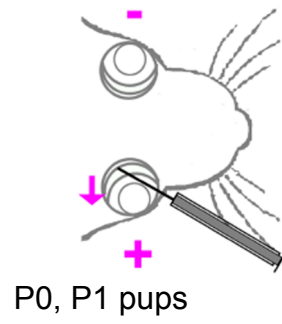

**B**

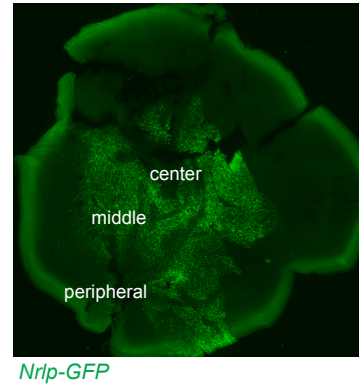

Supplement: Supplementary file 1 [file life-14-01103-s001.zip › Supplemental FigS1 0317-2024.pdf]

Supplemental figure S2

**A**

M/S cone

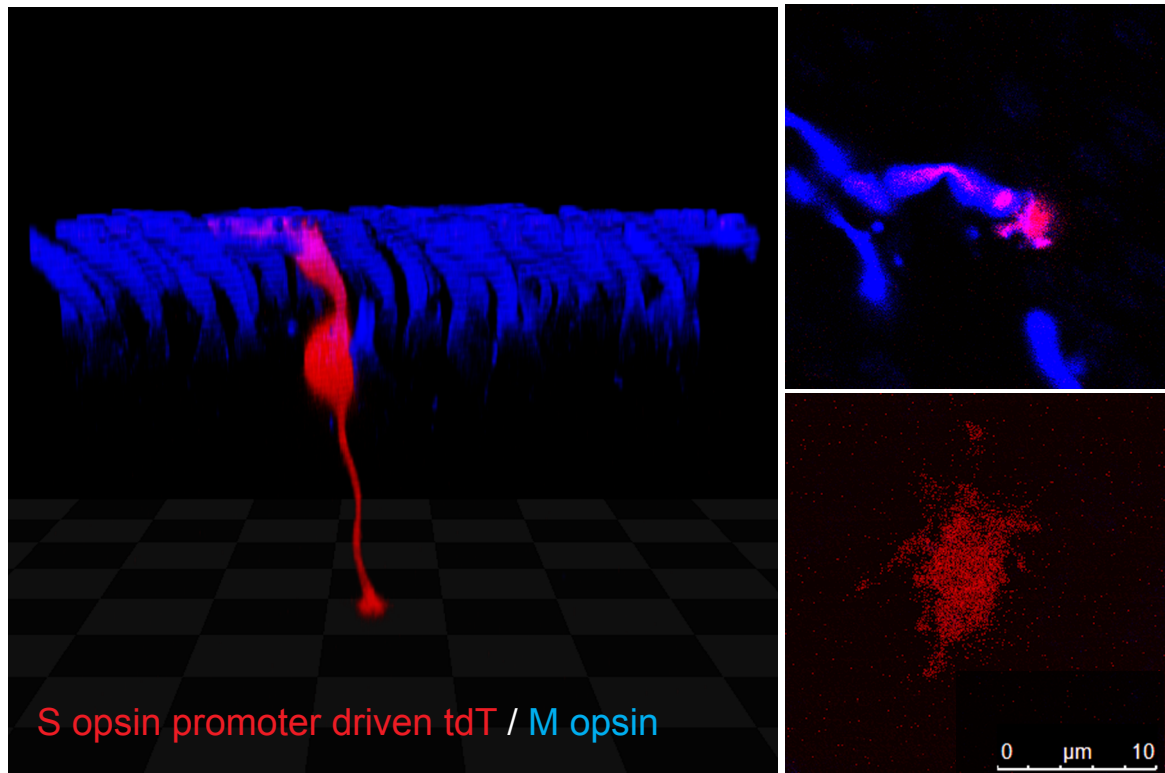

**B**

Pure S cone

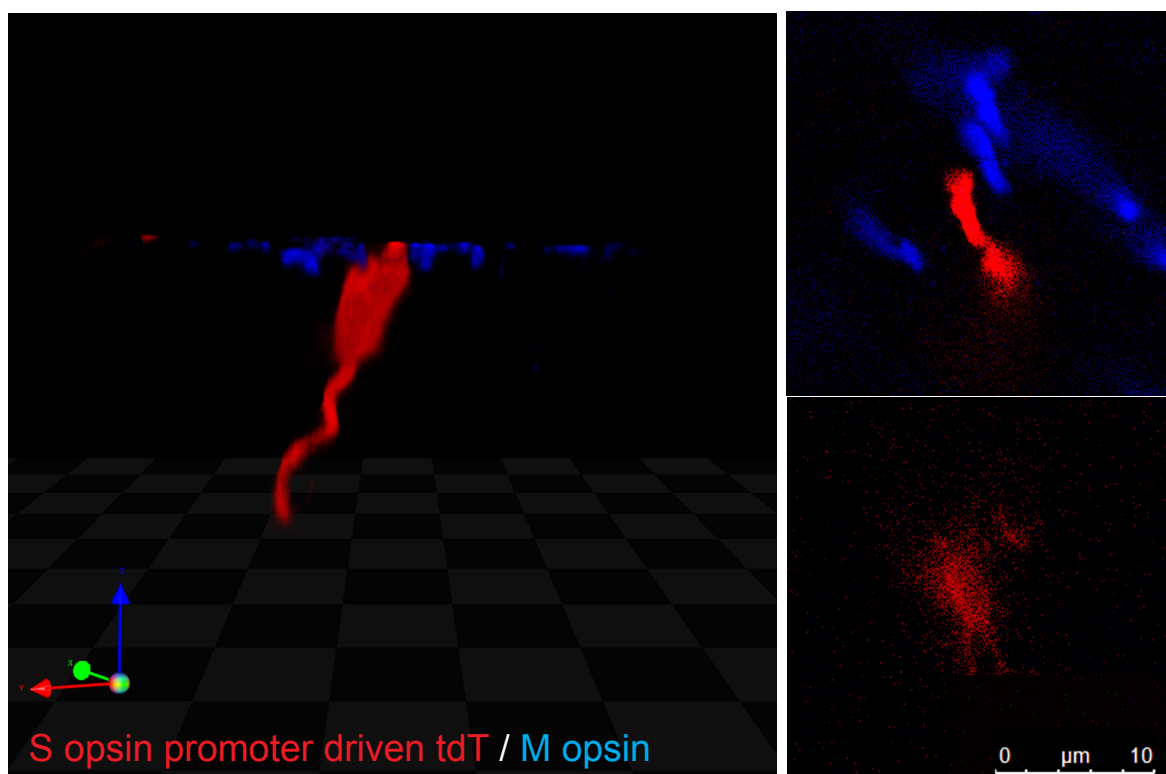

Supplement: Supplementary file 1 [file life-14-01103-s001.zip › Supplemental figS2 0317-2024.pdf]

Supplemental figure S3

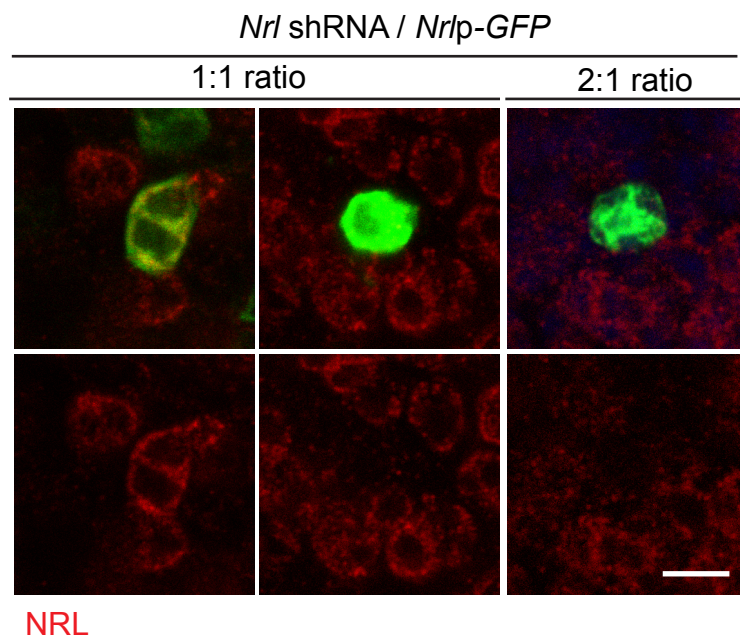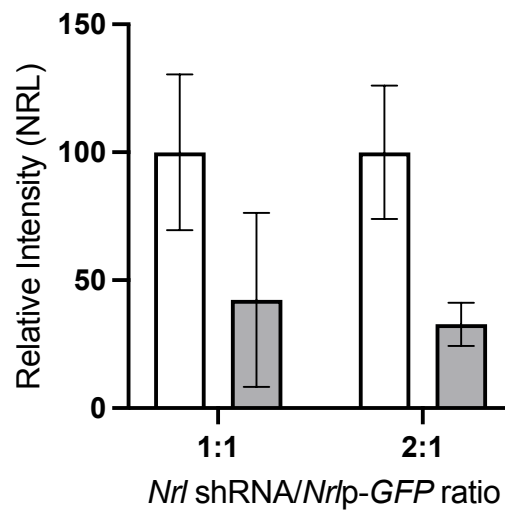

Ctrl (Green negative)  
*Nrl* shRNA/*Nrlp*-GFP (Green positive)

Supplement: Supplementary file 1 [file life-14-01103-s001.zip › Supplemental FigS3 0820-2024.pdf]

Supplemental figure S4

**A**

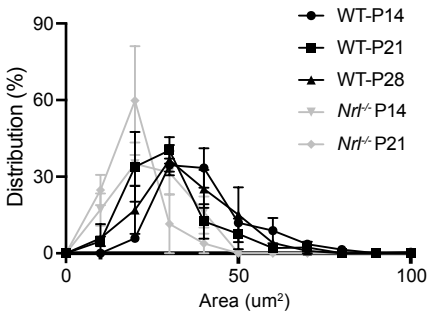

**B**

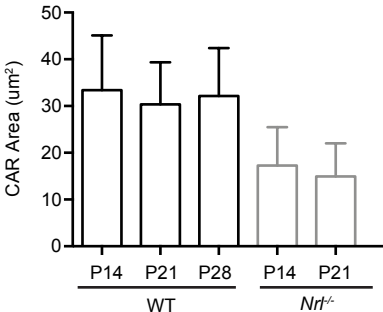

Supplement: Supplementary file 1 [file life-14-01103-s001.zip › Supplemental FigS4 0820-2024.pdf]

Supplemental Figure S5

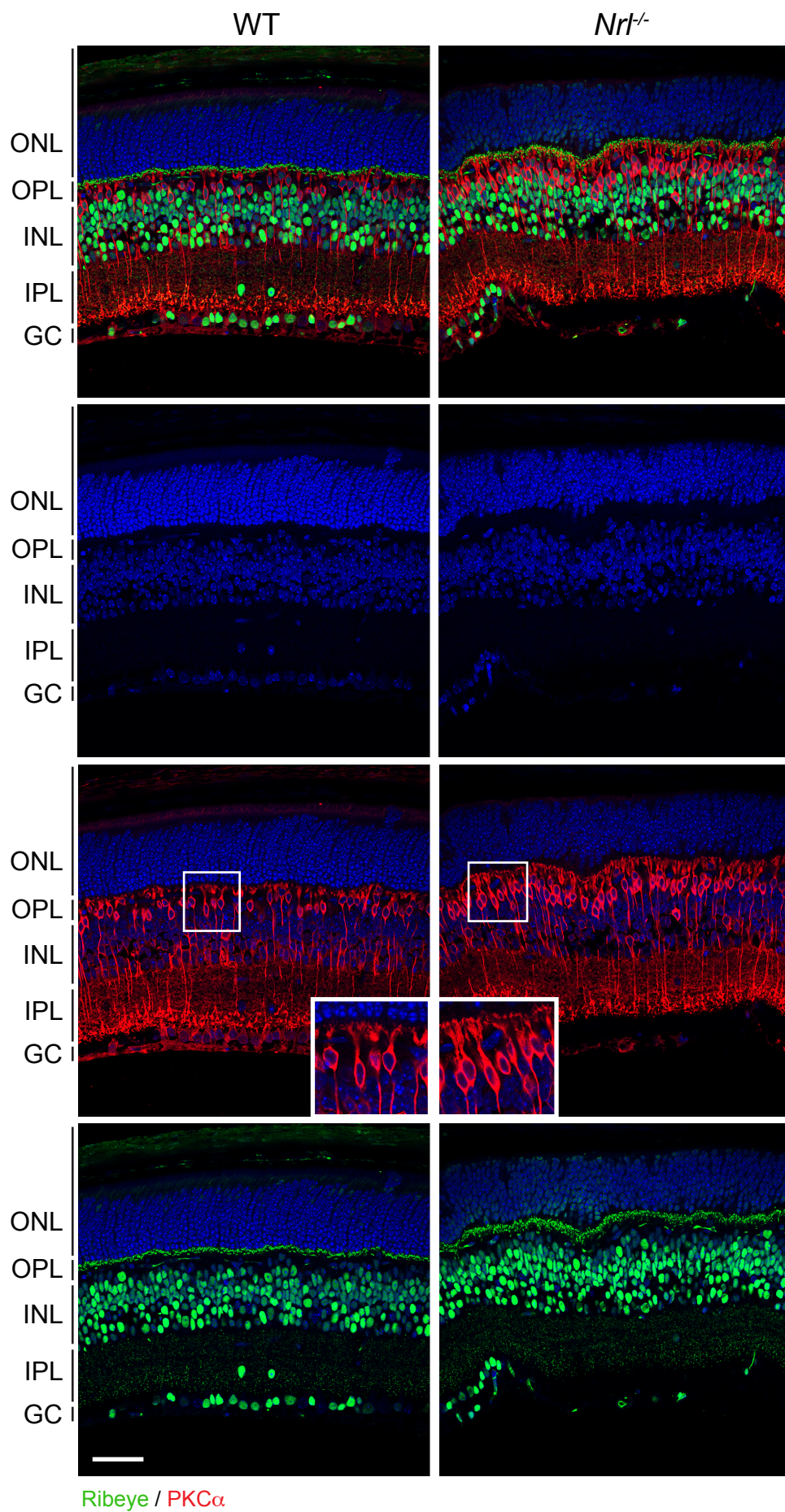

Supplement: Supplementary file 1 [file life-14-01103-s001.zip › Supplemental FigS5 PKC a.pdf]

Supplemental figure S6

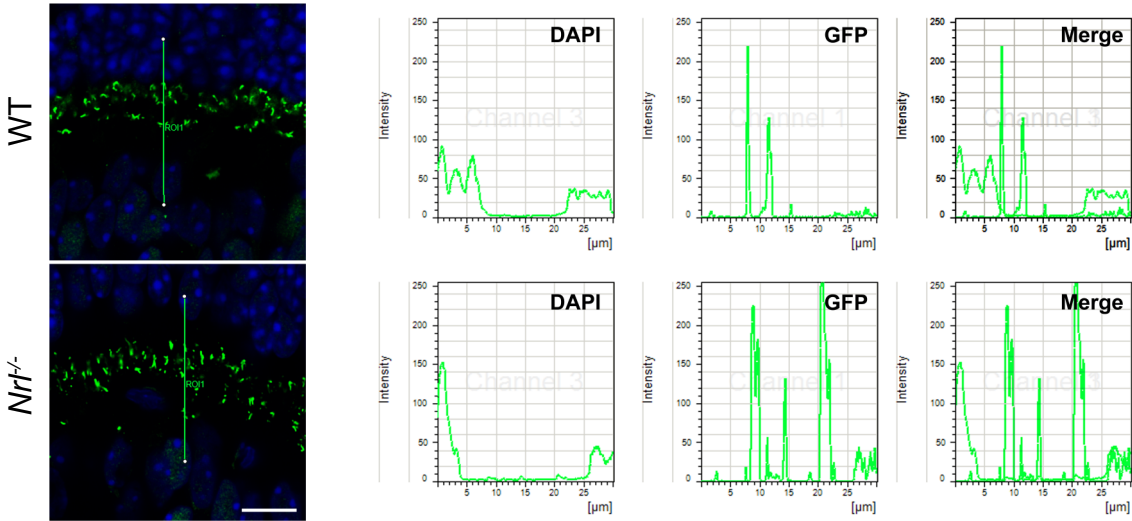

Supplement: Supplementary file 1 [file life-14-01103-s001.zip › Supplemental FigS6 0317-2024.pdf]

Supplemental figure S7

**A**

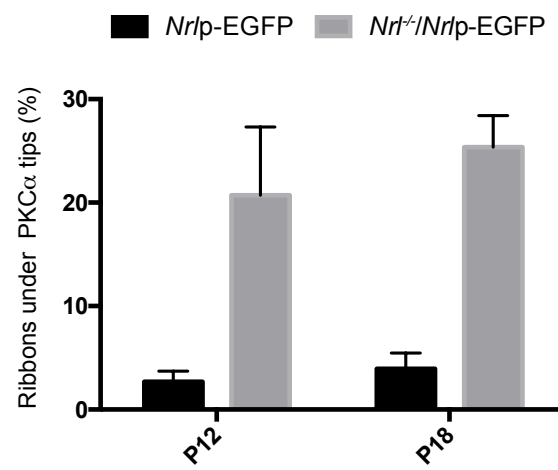

**B**

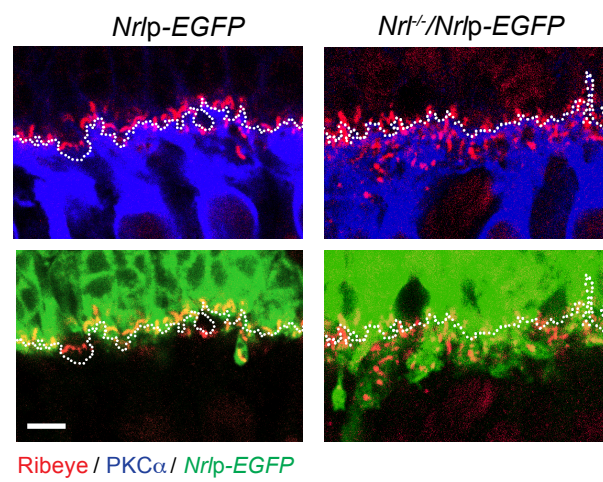

Supplement: Supplementary file 1 [file life-14-01103-s001.zip › Supplemental FigS7 0820-2024.pdf]

Supplemental figure S8

A

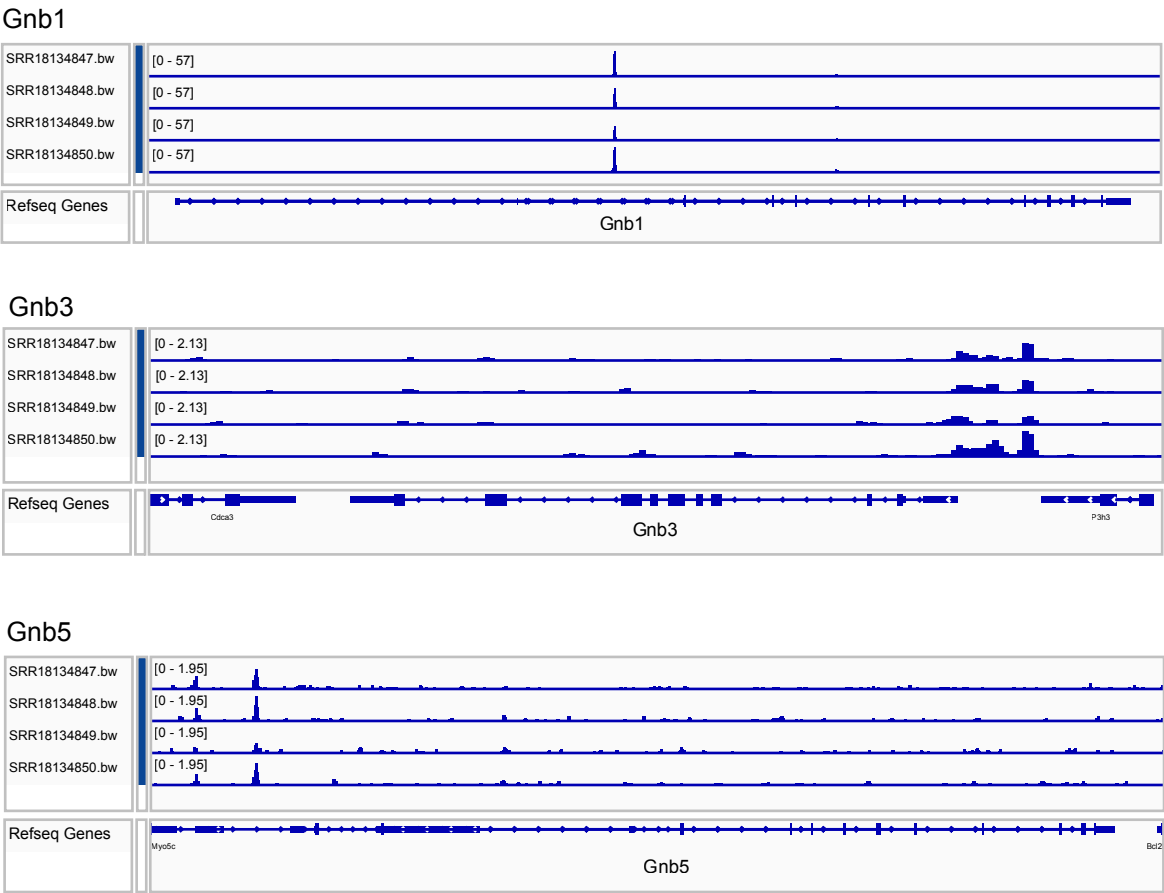

B

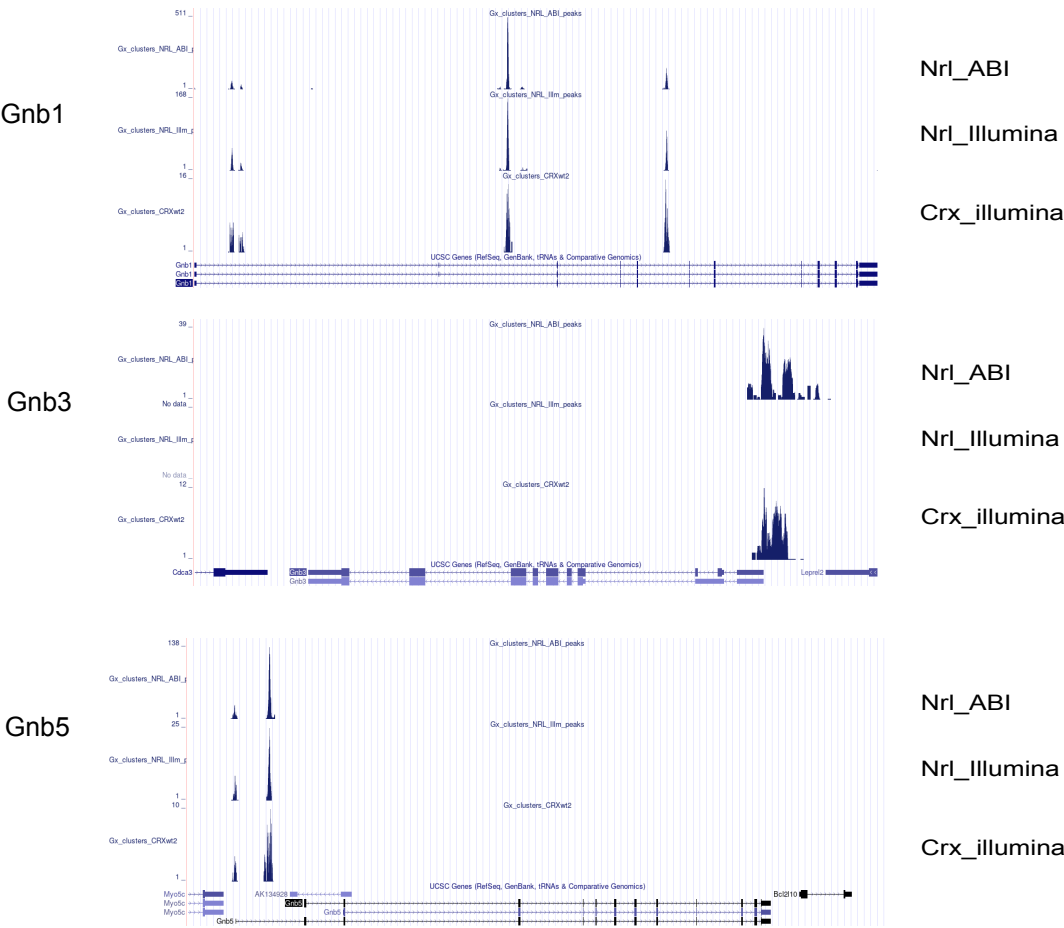

Supplement: Supplementary file 1 [file life-14-01103-s001.zip › Supplemental FigS8 0317-2024.pdf]
